# Barriers to and Motivations for the Implementation of a Treatment Programme for Latent Tuberculosis Infection using Isoniazid for People Living with HIV, in Upper Northern Thailand

**DOI:** 10.5539/gjhs.v5n4p60

**Published:** 2013-03-26

**Authors:** Saiyud Moolphate, Saranath Lawpoolsri, Petchawan Pungrassami, Natpatou Sanguanwongse, Norio Yamada, Jaranit Kaewkungwal

**Affiliations:** 1 Department of Tropical Hygiene, Faculty of Tropical Medicine, Mahidol University, Thailand; 2 Research Institute of Tuberculosis, Japan Anti-Tuberculosis Association (RIT-JATA), Japan; 3 Bureau of Tuberculosis, Department of Disease Control, Ministry of Public Health, Thailand; 4 TB/HIV Research Foundation, Thailand; 5 National AIDS Committee, Department of Disease Control, Ministry of Public Health, Thailand

**Keywords:** TB, HIV, IPT, isoniazid preventive therapy, barrier, motivation, Thailand

## Abstract

**Background:**

Isoniazid Preventive Therapy (IPT) has been recommended by WHO/UNAIDS for people living with HIV (PLWH) since 1993; however the uptake of IPT implementation has been very low globally. This study aims to assess the barriers to and motivations for the implementation of IPT for PLWH in upper northern Thailand, an area with a high tuberculosis (TB) and human immunodeficiency virus (HIV) burden.

**Methods:**

A survey was carried out via self-administered questionnaires mailed to healthcare workers (HCW) in all 95 public hospitals in the upper northern region of Thailand. A reminding phone call, one month after sending the mail, was made.

**Results:**

The response rate from the hospitals was 94% and from the HCW's, 70%. IPT programme was being implemented at only 18 (20%) out of the 89 public hospitals. The main barriers as reported by 144 HCWs working in hospitals without IPT programme, were: (1) unclear direction of national policy (60%), (2) fear of emerging Isoniazid resistant tuberculosis (52%), and (3) fear of poor adherence (30%). The 38 HCWs from hospitals implementing IPT programme, were motivated by (1) knowledge that IPT can prevent TB (63%), (2) the following of national guideline (34%), (3) concern for TB prevention even after the expansion of access to antiretroviral therapy (ART) (32%).

**Conclusion and Recommendation:**

To implement an IPT programme for PLWH, giving a clear national policy and straightforward direction are necessary. Furthermore, provision of public health information and updated evidences may enhance HCW's comprehension of benefits and risks of IPT, thus it may increase the IPT programme implementation.

## 1. Introduction

Currently, TB-HIV co-infection is the leading cause of death among people living with HIV (PLWH), bringing about a persistently high mortality rate across the world (Aung et al., 2013; Au-Yeung et al., 2011; WHO, 2012). TB is the main cause of death in PLWH in high TB burden countries such as Thailand (Ansari et al., 2002; Kantipong, Murakami, Moolphate, Aung, & Yamada, 2012; Straetemans, Bierrenbach, Nagelkerke, Glaziou, & van der Werf, 2010). Therefore, urgent action for the prevention of tuberculosis among PLWH, remains a high priority public health issue, both nationally and globally. Treatment of latent tuberculosis infection (TLTI), or Isoniazid preventive therapy (IPT), can prevent the progression of latent TB infection to active TB in PLWH by 33 % in overall and by 64% in tuberculin skin test positive group (Akolo, Adetifa, Shepperd, & Volmink, 2010). Moreover, observational studies in Brazil, South Africa and northern Thailand has reported the effect of IPT and IPT added on antiretroviral therapy (ART) on preventing TB and reducing the mortality (Charalambous et al., 2010; Golub et al., 2009; Golub et al., 2007; Moolphate, 2012).

In 1993 World Health Organization (WHO) outlined the strategies for TB prevention in PLWH via a policy statement on preventive therapy against tuberculosis (WHO/IUATLD, 1993). The policy was revised in 1998 (WHO/UNAIDS, 1998) and again in 2011 (WHO, 2011). In an attempt to respond to the TB/HIV syndemic and decrease the burden of tuberculosis in PLWH, WHO set up a new strategic framework in 2004-the “Interim policy on collaborative TB/HIV”. The strategy entitled “Three Is for HIV/TB” included the following strategies: Intensification of TB case-finding, Isoniazid Preventive Therapy, and Infection control (WHO, 2004).

Despite the WHO recommendation for IPT to PLWH, since 1993, implementation has been very low globally, including Thailand. In 2009, only 1.3% of PLWH globally received IPT (WHO, 2010); in 2011, only 8 out of 41 high TB/HIV burden countries (19.51%) reported providing IPT for PLWH in WHO global tuberculosis report (WHO, 2012). IPT has mostly been implemented in countries in South Africa, whilst in Southeast Asia, despite the fact that a large number of countries have a high HIV prevalence, only a few countries have undertaken to implement IPT programme. Consequently, scarce literature existed for IPT in Asia. Most previous studies assessing barriers to IPT implementation have been conducted in African countries. The results from such studies, however, may not be directly applicable to Asian setting, given that health systems and burden of the diseases differ so much from region to region.

In Thailand, IPT was recommended for the first time in a national TB/HIV guideline entitled “National recommendation guideline: the integrated TB/HIV strategies for the control and prevention of tuberculosis in Thailand” in 2003 (Thailand Ministry of Public health, 2003). That TB/HIV guideline was revised in 2005 and 2008 (Thailand Ministry of Public Health, 2005, 2008). In the last version of TB/HIV guideline 2008, IPT was recommended as an option for implementation (Thailand Ministry of Public Health, 2008). In the upper northern of Thailand, the manual for the IPT in PLWH had been made by office of communicable disease control region 10, Chiang Mai, Thailand since 2001 (Communicable Disease Control Region10 Thailand, 2001). In 2011, WHO listed Thailand as one of the top 22 high global TB burden countries, and one of the top 41 high global TB/HIV burden countries (WHO, 2012). IPT represented one element of the collaborative TB/HIV activities recommended by WHO to the high TB/HIV burden countries. Thailand, therefore, was expected to implement such an IPT programme. However it did not report about providing IPT for PLWH recently in WHO global tuberculosis report (WHO, 2012).

A nation-wide cross-sectional study surveyed the doctor's adherence to WHO IPT guideline in Thailand in 2002 (Hiransuthikul et al., 2005). It was conducted before Thai national recommendation guideline of IPT in Thailand. However, no study has been yet conducted after launching Thai IPT guideline. Since the programme uptake was slow, it is important to identify the key barriers to implementation of IPT. Furthermore, it would be worthwhile to study the factors that motivated the implementation staffs, healthcare workers, at the regional, provincial and district level hospitals where IPT programme was successfully carried out.

The northern region is one of the four regions of Thailand. Northern Thailand is a particularly good example, given its reported high burden of tuberculosis and HIV, their related mortality and its long history of IPT programme (Moolphate et al., 2011; Jittimanee et al., 2007; Payanandana, 1999). Northern region of Thailand comprises 17 provinces and 8 out of those provinces form upper northern Thailand. HIV prevalence among 21-year-old military conscripts in upper northern of Thailand was 0.9% in 2011. HIV prevalence among female sex worker was 4.96% in northern region of Thailand in 2011 (Bureau of Epidemiology Thailand, 2011) whilst the estimated national HIV prevalence of Thailand in 2011 was 1.2% (UNAIDS, 2011). TB notification rate of new case in all form of tuberculosis in Thailand was 92 per 100,000 populations in 2011 and 15% of TB patients were HIV infected (WHO, 2012). TB notification rate of new cases in all form of tuberculosis of upper northern Thailand was 90 per 100,000 in 2011 and 18% of TB patients were HIV infected (Disease Prevention Control 10 Thailand, 2012). A research study assessing barriers to and motivations for IPT implementation would benefit prevention of TB among PLWH in high burden setting.

Such a study is a necessary gap which current research has attempted to fill. By identifying and removing the major barriers to IPT implementation in an area such as this, IPT programmes can be put into place and replicated elsewhere. This study aimed to find out the barriers to IPT implementation, as well as discover the motivating factors for existing IPT programmes, within the upper northern region of Thailand.

## 2. Method

### 2.1 Study Design, Study Setting and Study Population

A descriptive cross-sectional mail survey was conducted during July to September 2012. The study site was upper northern region of Thailand, an area with a high TB and HIV burden as mentioned earlier. All the 95 public hospitals under Ministry of Public Health administration in that region were sampled, comprising 86 community hospitals, 7 general hospitals and 2 regional hospitals. TB and HIV clinics in those hospitals are headed by chief nurses. There is at least a physician taking care of HIV and/or TB patients in every hospital. Thus a physician in charge of TB and HIV care, HIV clinic nurse and TB clinic nurse from each hospital were considered as sampling elements. The questionnaires were mailed to those hospitals stating three targeted respondents to answer the survey questionnaires and mail back to the researcher. The participation was requested with an informed consent form to sign by each participant.

### 2.2 Data Collection Tool, Survey Administration and Ethics

The questionnaire used to assess barriers to IPT implementation was devised according to the WHO framework of six health system components, relating to: (1) leadership and governance, (2) service delivery, (3) supplies and products, (4) health workforce, (5) heath information system, and (6) health system financing (Getahun et al., 2010; WHO, 2007). The content validity was assessed by 3 experts from within the TB/HIV field. The questionnaire was tested and modified two times in order to enhance the comprehensibility of self-administered questionnaires for the respondents.

There were two parts to the questionnaire-the first part was for the hospitals without an IPT program in place, and the second part was for the hospitals with an IPT program in place. Each part consisted of 3 sections. The first section was a checklist of pre-specified items, including one open item for respondents to select in case the item list didn’t include their choice. Each respondent was asked to select, without ranking, 3 main reasons for either the barriers to or motivation for IPT. The second section asked respondents to provide “agree” or “disagree” answers to questions relating to the six health system components. This part was applied to nurse only. The third section was made up of open-ended questions allowing respondents to give comments and suggestions regarding the policy (feedback from field to policy).

The Ethics Committee of the Faculty of Tropical Medicine, Mahidol University, Thailand, approved this study (Reference number NUTM 2012-019-01). The Provincial Health Office Chief of each of the 8 study provinces approved the collection of information from the hospitals under their responsibility. Furthermore, permission from the directors of each hospital was also obtained. A set comprising a cover letter, explaining the objective of the survey, an ethics committee approval letter, a letter of approval from the Provincial Health Office Chief, a participant information sheet, a consent form, a questionnaire and a TB-logo magnet, was sent to each of the 95 hospitals. This set was then passed on to the physicians and nurses following approval from the director. An envelope and stamps were enclosed for the convenient return of mail. No payments were made to study participants who answered the questionnaire, only a TB-logo magnet was given. The reminding phone call, one month after sending the mails, was made to the nurse.

### 2.3 Data Analysis and Sample-Power Calculation

The double entry system was applied to save the data via electronic copy. Validation of two data sets was performed. Descriptive statistics, such as percentages, mean or median, standard deviation or interquartile range, were used to summarize the data. Data analysis applied STATA version 11.

All 95 hospitals under Ministry of Public Health administration in upper northern of Thailand were considered as study population size. Using Krejcie and Morgan's formula for category data (Kotrlik & Higgins, 2001; Krejcie & Morgan, 1970), a minimum required sample of 76 hospitals would be enough for 95% confidence level and 5% of acceptable error.

## 3. Results

### 3.1 Study Participants and Response Rate

The questionnaire survey was sent to 95 hospitals, of which 89 hospitals agreed to participate, giving a response rate of 94%. One hospital refused to participate following a decision from its own institutional review board. Within the 94 hospitals, the questionnaires were given to 282 health care workers, out of which 198 agreed to participate in the survey. The overall response rate from the health care workers was 70%; from the TB clinic nurses it was 81%; from the HIV clinic nurses it was 83%; and from the physicians it was 47%.

Of the 198 respondents (Table 1), 22% were physicians, 38% were TB clinic nurses and 39% were HIV clinic nurses. 73% of the respondents were female. The mean age of all respondents was 40 years (standard deviation 8.6 years). The median time of work experience was 17 years for the TB clinic nurses, 20 years for the HIV clinic nurses and 7 years for the physicians. 50% of the physicians had never seen the Thai national TB/HIV collaborative guidelines and 45% of them did not know that IPT guidelines were outlined in the TB/HIV collaborative guideline book. In contrast, only 11% of TB clinic nurses and 13% of HIV clinic nurses had never seen the Thai national TB/HIV collaborative activities guidelines. Approximately 12% of the TB clinic nurses and 21% of the HIV clinic nurses did not know that IPT guidelines were outlined in the TB/HIV collaborative guideline book. Most of the health care workers (77%) had never seen the WHO IPT guidelines.

**Table 1:** Characteristics of respondents of the mailing cross-sectional survey

Characteristic Number	TB nurse	%	HIV nurse	%	Physician	%	Total	%
	76		78		44		198
**Sex**									
Male	17	22.4	10	12.8	26	59.1	53	26.8
Female	59	77.6	68	87.2	18	40.9	145	73.2
**Age**									
Mean (year)	40		42		35		40	
Standard deviation (year)	±8.1		±7.8		±8.8		±8.6	
**Government-service experience**									
Median (year)	17		20		7		17	
Interquartile (year)	11-22		16-27		2-14		10-23	
**Have you ever seen the Thai national TB/HIV collaborative activities guidelines?**									
Never	8	10.5	10	13.2	22	50.0	40	20.4
Yes, but not used in practice	20	26.3	22	28.9	12	27.3	54	27.6
Yes, and used in practice	48	63.2	44	57.9	10	22.7	102	52.0
**Did you know that IPT guidelines are outlined in the TB/HIV collaborative guideline book?**									
No	9	11.8	16	20.8	20	45.5	45	22.8
Yes	67	88.2	61	79.2	24	54.6	152	77.2
**Have you ever seen the IPT guidelines for Northern Region 10 ?**									
Never	46	61.3	48	63.2	35	79.5	129	66.2
Yes, but not used in practice	8	10.7	12	15.8	3	6.8	23	11.8
Yes, and used in practice	21	28.0	16	21.1	6	13.6	43	22.1
**Have you ever seen the WHO IPT implementation guidelines?**									
Never	56	75.7	65	84.4	29	67.4	150	77.3
Yes, but not used in practice	9	12.2	8	10.4	10	23.3	27	13.9
Yes, and used in practice	9	12.2	4	5.2	4	9.3	17	8.8

### 3.2 The Coverage of IPT Implementation in Hospitals in Upper Northern Thailand

**Figure 1:**
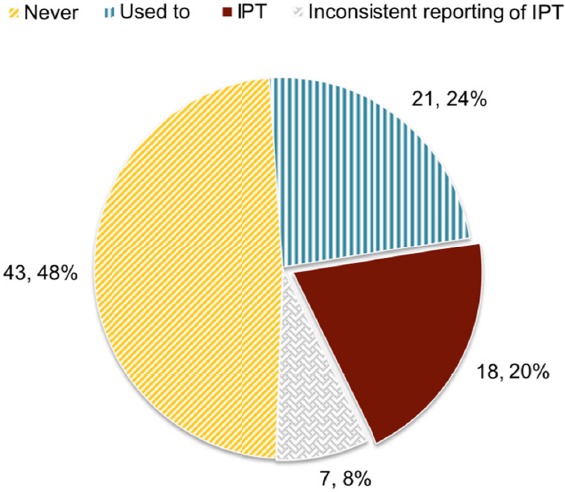
The coverage of IPT implementation in hospitals in upper northern Thailand

According to the responses of 89 hospitals in upper northern Thailand (Figure 1), 18 hospitals (20%) were shown to be currently implementing IPT for PLWH. IPT programmes had been implemented in another 21 hospitals (24%), although these programs had been cancelled. A further 43 hospitals (48%) were shown to have never implemented IPT for PLWH. It should be noted that there was inconsistent reporting from respondents regarding IPT implementation in the remaining 7 hospitals (8%).

### 3.3 Barriers to Providing IPT to People Living with HIV

#### 3.3.1 Barriers to Providing IPT

From the 64 hospitals without IPT programmes, a total of 144 healthcare workers reported on the major barriers to IPT implementation (Table 2). According to these healthcare workers, the main barrier to IPT implementation was caused by unclear direction of national policy (60%); the second reason was due to a fear of Isoniazid (INH) resistance (52%); and the third reason was due to a fear of poor adherence by health care workers (30%). It is interesting to note that, according to the physicians’ opinion only, the main barrier to IPT implementation was due to a fear of generating INH resistance (81%). This physician's opinion was significantly different from nurses’ opinion (Table 2). Moreover, the proportion expressing concern for workload of health care worker was significantly larger among the nurses than among the physicians (Table 2).

**Table 2:** Barriers to providing IPT to people living with HIV

Reason not to implement IPT	Total (n=144)	TB and HIV Nurses (n=113)	Physicians (n=31)	P-value
IPT implementation was not clear direction in national policy	86 (60%)	65 (58%)	21 (68%)	0.36
Fear of Isoniazid resistance	75 (52%)	50 (44%)	25 (81%)	0.00
Fear of poor adherence by health care worker	43 (30%)	33 (29%)	10 (32%)	0.79
Difficulty with the administration of tuberculin skin test	35 (24%)	29 (26%)	6 (19%)	0.44
Workload of health care worker	27 (19%)	26 (23%)	1 (3%)	0.01
Fear of toxicity of Isoniazid	26 (18%)	18 (16%)	8 (26%)	0.22
Most HIV-infected patients already undergoing Antiretroviral therapy	25 (17%)	21 (19%)	4 (13%)	0.44
Unsure about the effectiveness of IPT	24 (17%)	15 (13%)	9 (29%)	0.04
IPT has a short term benefit	18 (12%)	15 (13%)	3 (10%)	0.57
IPT does not provide a survival benefit	3 (2%)	2 (2%)	1 (3%)	0.63
Others	26 (18%)	25 (22%)	1 (3%)	0.01

*Note: 1. The percentage was calculated from (Number of person mentioning reason/Number of respondent of each reason)*.

*2. Each respondent was asked to select 3 main reasons out of 11*.

*3. Excluded 15 respondents from hospitals that reported inconsistent answers of IPT implementation*.

#### 3.3.2 Barriers against Provision of IPT based on 6 WHO Health System-Related Components

The questionnaires on the barriers to IPT implementation according to the WHO framework of six health system components were completed by 113 nurse respondents (Table 3.). Agreement was shown to be highest for the component relating to leadership and governance (82%), stating that the direction of national policy towards IPT implementation was unclear. Consensus on the other 5 components was approximately half: service delivery-related (57%); supplies and products-related (66%); health system financing-related (62%); health information system-related (61%); and health workforce-related (60%).

**Table 3:** Barriers against provision of IPT based on 6 WHO health system-related components

Description	Number of respondents	Agree	Percentage (%)
**1. Leadership and governance-related**			
Not clear direction of IPT implementation by Bureau of TB	99	76	77
Not clear direction of IPT implementation by Bureau of AIDS	97	75	77
IPT implementation was not supported by some experts	94	46	49
No IPT due to lack of support from Northern Region CDC 10	93	32	34
No IPT due to lack of support from Provincial Health Office	94	30	32
No IPT due to lack of support from hospital physician	92	28	30
No IPT due to lack of support from hospital director	97	23	24
Summary: No IPT due to a lack of clear nation policy for IPT implementation	97	80	82

**2. Service delivery-related**			
No IPT due to fear of poor adherence	101	57	56
No IPT due to difficulty with the administration of tuberculin skin test	101	49	49
No IPT due to lack of operation guidelines or details for IPT provision	84	34	41
No IPT due to PLWH refusing to take IPT	99	32	32
Summary: No IPT due to difficulty with service delivery of IPT	102	58	57

**3. Supplies and products-related**			
No IPT due to difficulty with managing Purified Protein Derivative (PPD) as it needs cold chain management	97	69	71
No IPT due to unavailability of PPD	98	40	41
No IPT due to lack of support for isoniazid drug	99	15	15
Summary: No IPT because of difficulties with supplies and products	96	63	66

**4. Health system financing-related**			
No IPT due to doubt about cost-effectiveness of IPT programme	98	65	66
No IPT due to lack of extra central budget support for IPT implementation	100	63	63
Summary: No IPT because of lack of health system financing	98	61	62

**5. Health information system-related**			
No IPT due to lack of monitoring and supervision from national bureaus of TB and AIDS	99	62	63
No IPT due to not having standard IPT form or report from national bureaus of TB and AIDS	100	47	47
Summary: No IPT due to problems relating to health information system	100	61	61

**6. Health workforce –related**			
No IPT due to lack of staff to monitor and evaluate IPT programme at national level	100	79	79
No IPT due to lack of training for HCW resulting in a lack of confidence in providing IPT	100	51	51
No IPT due to lack of clear responsibilities between HIV HCW’s and TB HCW’s	101	42	42
No IPT due to workload of HCW	101	41	41
**Summary: No IPT due to difficulties relating to human resources**	**101**	**60**	**60**

*Note: 1. Excluded 15 respondents from hospitals that reported inconsistent answers of IPT implementation*.

*2. The information in this table was obtained from the nurses in TB and HIV clinics only*.

### 3.4 Motivation for Providing IPT to People Living with HIV

The major motivations for implementing IPT were given by 38 health care workers (9 physicians and 29 nurses) currently working in hospitals with existing IPT programmes. The main reasons for implementing IPT programmes were: (1) knowledge that IPT can prevent TB (63%); (2) Following of national guidelines regarding IPT implementation (34%); (3) concern that even though PLWH received ART, TB can still develop and needs to be treated for latent tuberculosis infection (32%). The reasons are shown in Table 4.

**Table 4:** Motivations for providing IPT to people living with HIV

Reasons	Number of person mentioning reason (n=38)	Percentage (%)
IPT can prevent TB	24	63
Following of national guidelines regarding IPT implementation	13	34
Concern that TB can still develop in PLWH receiving antiretroviral therapy and so needs to be treated to prevent TB by IPT	12	32
Effective counselling and education can be done to improve patient adherence	11	29
Toxicity of INH is low compared to the benefit of IPT for PLWH	8	21
IPT provides a survival benefit	7	18
No evidence of IPT causing increased drug resistance	5	13
Supported by research organizations	4	11
Other	3	8

*Note: 1. Each respondent was asked to select 3 main reasons out of 9*.

*2. Excluded 15 respondents from hospitals that reported inconsistent answers of IPT implementation*.

## 4. Discussion

According to WHO TB/HIV collaborative guidelines, IPT is one of the 3I's strategy and should be implemented in northern Thailand. In this survey, IPT programme implementation was reported in only 18 out of 89 public hospitals (20%). Many hospitals (72%) have not implemented IPT, the main reasons being: unclear direction of national policy (60%), fear of INH resistance (52%), and fear of poor adherence (30%).

### 4.1 Barriers to IPT Implementation

As it was found that the main barrier to IPT implementation was leadership and governance-related, it could be argued that the direction of national policy from both the Bureau of Tuberculosis and the Bureau of AIDS was not clearly stated to the hospitals. Having a policy or set of guidelines for IPT, but experiencing a lack of implementation, is common in many countries. A cross-sectional e-mail survey by WHO reported that only 28% of the countries with a national policy for IPT had achieved nationwide implementation (Date et al., 2010).

The current study evidence shows that among the 44 doctors surveyed, only 33%, working at various hospital levels, have seen the WHO IPT guidelines. About half of them have never seen the Thai national TB/HIV collaborative activities guidelines or knew that IPT guidelines were included in the national TB/HIV collaborative strategies. Only 29% of them have prescribed IPT to PLWH. In 2002 a national survey of Thai physicians reported that 61% of them had seen the WHO guidelines concerning IPT, whereas only 19% of them implemented IPT (Hiransuthikul et al., 2005). The guidelines were not sufficiently put into practice. Thus the existence of national guidelines was not a guarantee of implementation in practice (Grimshaw et al., 2004). Moreover, a qualitative study of 22 health care workers in South Africa reported that some expert clinicians even opposed IPT, leading to increased reluctance within hospitals to prescribe IPT (Lester et al., 2010).

Among the barriers answered by HCWs, the fear of generating INH resistance stood out in second. There is common consensus that active TB is difficult to detect among PLWH. Hence, many physicians in limited-facility settings are hesitant to prescribe IPT to PLWH. Whilst “fear of generating drug resistance” was the second most common barrier perceived amongst respondents as a whole, and it was the most common barrier perceived by the physicians (Table 2). This finding was concurrent with the result of an email cross-sectional survey conducted by WHO in 69 high burden countries in 2007 (Date et al., 2010). It stated that “fear of generating drug resistance” was the most common answer and the main reason for not giving IPT to PLWH, despite considering inadequate TB case finding and difficulties in excluding active TB. Moreover, a Thai national survey in 2002 also reported physicians’ concern about the inducement of INH resistance, as a reason for not providing IPT (Hiransuthikul et al., 2005). A qualitative study of HIV clinic staff in South Africa also reported a lack of healthcare workers belief in the accuracy of TB screening, as the barrier to IPT implementation (Lester et al., 2010).

There is a point of controversy between recent WHO IPT guidelines for starting IPT and the opinion of physicians in practice. WHO IPT Guidelines 2011 stated that a chest X ray was not compulsory before starting IPT (WHO, 2011). However, the current study results have shown that all respondent physicians disagree with this (data not shown).

A systematic review, assessing the effect of IPT on the risk of INH resistant TB, reported that IPT increases the risk of INH resistance by 1.45 times, whilst it was not significant (Relative risk 1.45; 95% confidence interval 0.85-2.47). However, it should be noted that analyses were limited because of small samples and, moreover, the relative risk does not exclude an increased risk for INH resistant TB (Balcells, Thomas, Godfrey-Faussett, & Grant, 2006). Therefore, effective TB screening tools and adequate diagnostic facilities, to exclude active TB cases and identify IPT candidates, would be required to reinforce healthcare worker’ belief that the implementation of IPT is well-structured and safe.

The third most commonly answered barrier was fear of poor adherence to IPT. IPT adherence is a patient-derived barrier. It is a common reason also relating to health service delivery. It is difficult to ensure adherence to the complete nine months long programme of INH therapy. The adherence rate has been variable across different settings, in previous reports. Adherence to IPT has fluctuated from 47% to 94% both in randomized controlled trials and observational studies (WHO, 2011). According to unpublished data, the IPT treatment completion rate in Chiang Rai province, northern Thailand, ranged from 82% to 93% in the period 2003 to 2009.

This reason, of fear of poor adherence to IPT, was found to be the second or third commonest reason in most previous studies. A previous survey in Thailand reported that it was the second most perceived barrier by the physicians in 2002. Similarly, WHO email survey in 2007 and current study consistently found it as third most perceived barriers of health care workers to implement IPT program (Date, et al., 2010; Hiransuthikul, et al., 2005). In contrast to those findings, a qualitative study in South Africa reported that the doctors were not too concerned about IPT adherence (Lester et al., 2010).

### 4.2 Motivations for IPT Programme Implementation

Most previous studies have assessed the reasons for not implementing IPT, but the current survey is the first to find out the reasons for implementing IPT. Among the 89 hospitals surveyed in upper northern Thailand, only 18 have implemented IPT programes. Characteristically, implementation of IPT in those 18 hospitals was carried out by an integrated collaboration between TB and HIV clinics. Most of the hospitals were 120 bed hospitals. IPT programs were started in those hospitals before the launch of the nationwide ART access policy. Among the eight provinces, Chiang Rai had the highest coverage of IPT. The 18 hospitals, which implemented IPT, reported strong rationales in their implementation decision. The principal motivation was due to healthcare worker knowledge of IPT. Of the HCW's working in hospitals with an IPT programme, 63% stated that they were aware of the fact that “IPT can prevent TB in PLWH”. This reflects the importance of healthcare workers’ knowledge and their clear understanding about translating guidelines into practice. Evidence from previous literature stated that doctors, who were unaware of, or uncertain of, IPT efficacy in preventing TB, were not willing to prescribe IPT (Lester et al., 2010). The current study findings have shown a positive point of view, it would be useful to introduce preventive intervention regarding IPT in hospitals. Furthermore, applying a positive deviance approach of these hospitals might serve as a good example for the implementation in the future of other hospitals which do not have experience for IPT implementation.

The second most common answer of motivation for IPT programme implementation, related to the following of national guidelines. Most of the respondents (69%) answered that they used the IPT manual, included in TB/HIV collaborative national guidelines (data not shown). An African study in 2011 reported that offering IPT was three times more likely where national guidelines were available. That cross sectional survey was conducted in 50 randomly selected health facilities in nine districts of South Africa in 2011; 35 (71%) of those participant health facilities had clearly stated that they followed national IPT implementation guidelines (Chehab, Vilakazi-Nhlapo, Vranken, Peters, & Klausner, 2012). It deviated from the finding of the previous multinational WHO email survey in 2007 even though two studies were conducted when new WHO 2011 IPT guideline was not yet in place (Date et al., 2010). Different study sites yielded different results suggesting the important need of finding in Southeast Asia, which current study had explored.

The third most common answer of motivation was of a concern for the prevention of TB, even after the expansion of access to ART. In the high TB burden setting of northern Thailand, the chance of TB infection is higher for PLWH at all times. There is strong evidence to show that implementation of IPT after the roll-out of ART significantly reduces the incidence of tuberculosis (Golub et al., 2009; Golub et al., 2007). This finding pointed out the importance of knowledge updating and awareness of IPT among health care workers.

## 5. Strengths and Limitations

The mailing survey was devised so as to face the common problem of an inadequate response rate. Several ways of strengthening the response rate, such as providing an attached request letter from the researcher to the respondent with a Mahidol University letter head, providing a stamp and envelope for returning mails, offering a souvenir magnet with a TB logo to remind the participants, and making a reminding phone call one month after sending the mails, were followed. These attempts resulted in a 94% response rate from hospitals and a 70% overall response rate from healthcare workers. However, one notable limitation was the response rate from physicians of only 47% even though we avoided asking lengthy questionnaires as in Table 3 to them.

## 6. Conclusion and Recommendation

Successful implementation of IPT programme within a country following WHO guidelines will logically depend on identifying and overcoming the barriers at national and regional level as well as motivation of implementers. Current study was an attempt seeking such critical information which would serve public health for programmatic prevention of tuberculosis among PLWH in upper northern Thailand and similar TB/HIV burdened settings.

In conclusion, less clear direction of national policy and health care worker's worry for emerging drug resistance strains were main barriers identified at the hospitals where IPT was not implemented, whereas clear knowledge on TB-preventive effect of IPT and following national policy motivated the health care workers at the hospitals where IPT was implemented.

Based on our findings, we recommend the national policy giving clear policy and straightforward direction for hospitals. Furthermore, providing updated information about the benefits and risks of IPT is importantly necessary to promote the motivation of HCWs leading to successful implementation of IPT programme.
